# Robust lumen segmentation based on temporal residual U-Net using spatiotemporal features in intravascular optical coherence tomography images

**DOI:** 10.1117/1.JBO.30.10.106003

**Published:** 2025-10-01

**Authors:** Mingrui He, Yin Yu, Kun Liu, Rongyang Zhu, Qingrui Li, Yanjia Wang, Shanshan Zhou, Hao Kuang, Junfeng Jiang, Tiegen Liu, Zhenyang Ding

**Affiliations:** a Tianjin University, School of Precision Instruments and Opto-Electronics Engineering, Tianjin, China; b Institute of Optical Fiber Sensing of Tianjin University, Tianjin Optical Fiber Sensing Engineering Center, Tianjin, China; c Tianjin University, Ministry of Education, Key Laboratory of Opto-electronics Information Technology, Tianjin, China; d State Administration for Market Regulation, Key Laboratory of Optical Fiber Sensing Metrology and Measurement, Tianjin, China; e International Joint Institute of Tianjin University, Fuzhou, China; f Chinese PLA General Hospital, Department of Cardiology, Beijing, China; g Nanjing Forssmann Medical Technology Co., Nanjing, China

**Keywords:** intravascular optical coherence tomography, deep learning, lumen segmentation, pullback spatiotemporal feature

## Abstract

**Significance:**

Lumen segmentation in intravascular optical coherence tomography (IVOCT) images is essential for quantifying vascular stenosis severity, location, and length. Current methods relying on manual parameter tuning or single-frame spatial features struggle with image artifacts, limiting clinical utility.

**Aim:**

We aim to develop a temporal residual U-Net (TR-Unet) leveraging spatiotemporal feature fusion for robust IVOCT lumen segmentation, particularly in artifact-corrupted images.

**Approach:**

We integrate convolutional long short-term memory networks to capture vascular morphology evolution across pullback sequences, enhanced ResUnet for spatial feature extraction, and coordinate attention mechanisms for adaptive spatiotemporal fusion.

**Results:**

By processing 2451 clinical images, the proposed TR-Unet model shows a well performance as Dice coefficient = 98.54%, Jaccard similarity (JS) = 97.17%, and recall = 98.26%. Evaluations on severely blood artifact-corrupted images reveal improvements of 3.01% (Dice), 1.3% (ACC), 5.24% (JS), 2.15% (recall), and 2.06% (precision) over competing methods.

**Conclusions:**

TR-Unet establishes a robust and effective spatiotemporal fusion paradigm for IVOCT segmentation, demonstrating significant robustness to artifacts and providing architectural insights for temporal modeling optimization.

## 1 Introduction

**Fig. 1:**
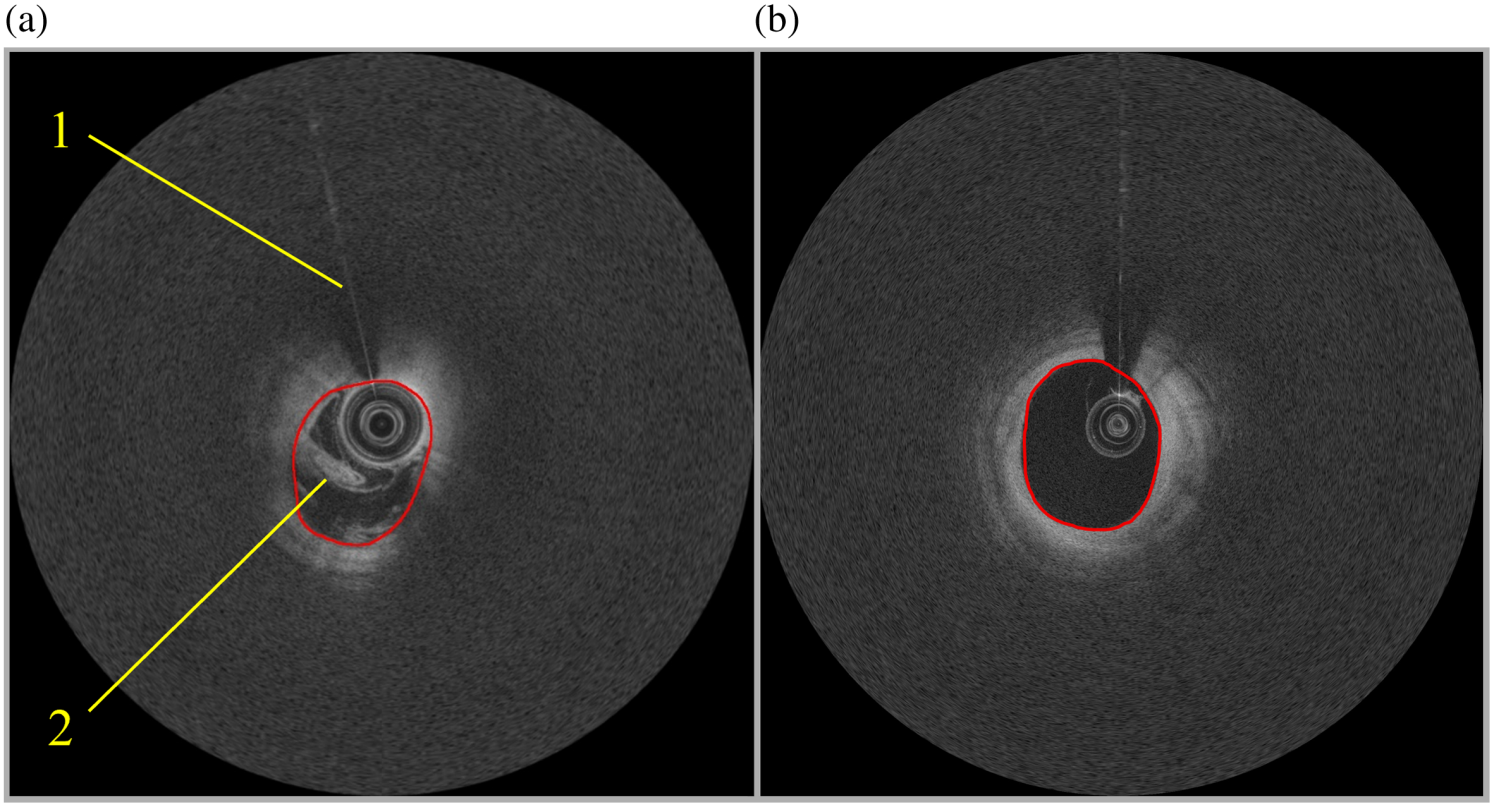
(a) IVOCT image with severe artifacts (1. Catheter artifact and 2. Blood artifact). (b) Well-imaged IVOCT image.

Cardiovascular disease refers to conditions affecting the circulatory system and is commonly caused by atherosclerosis. It is the leading cause of death worldwide and a significant contributor to health loss and excessive healthcare costs.^1^^,^^2^ Current imaging techniques for assessing coronary artery lumens include coronary angiography, intravascular ultrasound, intravascular optical coherence tomography (IVOCT), angioscopy, and near-infrared spectroscopy. Among these, IVOCT stands out as a high-resolution optical diagnostic technology capable of precisely evaluating the composition of coronary artery walls, identifying atherosclerotic lesion characteristics, and optimizing stent placement. It plays an irreplaceable role in improving precision and outcomes in coronary interventional therapy.^3^^,^^4^ The core of IVOCT imaging is based on the principle of fiber-optic Michelson interferometry. The typical clinical imaging procedure is as follows: First, an operator advances a catheter with an occlusion balloon and an integrated imaging wire into the target coronary artery. The balloon is then inflated to temporarily block blood flow proximal to the target lesion, whereas a continuous low-volume flush is administered to clear the imaging area of blood. Next, the imaging wire rotates rapidly and is pulled back automatically within the artery, capturing high-resolution cross-sectional images of the vessel wall. After imaging is complete, the balloon is deflated to restore blood flow, and the catheter and wire are withdrawn from the vessel. This process results in a series of continuous cross-sectional vessel images for clinical analysis. Lumen segmentation in IVOCT images is a crucial step for obtaining fundamental vascular measurements, including the severity, location, and length of vessel stenosis, as well as the extent of stent malapposition and restenosis burden.^5^ However, despite the high resolution and low noise of IVOCT images, challenges persist due to uneven grayscale distribution and the presence of artifacts, such as catheter-related and blood artifacts (Fig. 1). In addition, the variability in vascular structures and surrounding tissue compositions results in inconsistent appearances of vessel boundaries across different images. These issues often compel physicians to perform time-consuming manual corrections or entirely manual segmentation, which significantly prolongs the overall analysis process and may even lead to potential miscalculations of key morphological parameters—ultimately affecting treatment decisions. Therefore, automated lumen segmentation in IVOCT images remains a challenging task.

Traditional automatic image segmentation methods include edge-based,^6^ region-based,^7^ morphological feature-based,^8^ clustering,^9^ and surface-fitting approaches.^10^ To enhance segmentation accuracy, prior knowledge such as elliptical shape priors,^11^ connectivity priors,^12^ and star-shaped priors^13^ has been proposed to provide useful geometric information about the lumen region, mitigating the impact of poor imaging or blood-related artifacts.^14^^,^^15^ Among these, Ref. 16 proposed the uniqueness of vascular connected region (UVCR) method that demonstrates relative superiority under severe blood artifact conditions. Its core mechanism exploits the anatomical uniqueness of vascular walls along A-lines, identifying artifact-induced regions through detection of shared A-lines across multiple connected regions, combined with cross-validation of area ratios and spatial distributions. However, most traditional methods lack generalizability, requiring pre-set parameters based on prior experience and struggling to adapt to complex vascular environments. When the lumen contour is irregular or suboptimal images are encountered, these methods often demand extensive parameter adjustments or diverse preprocessing steps to minimize interference, limiting their efficiency and accuracy in segmentation.

In recent years, deep learning techniques have been widely applied in the field of medical imaging. For image recognition and classification, compared with traditional object/feature-based machine learning methods, deep learning can directly map raw input images to final classification results, avoiding inaccurate feature calculations and segmentation errors caused by subtle or complex objects. In the domain of image segmentation, Ref. 17 introduced the U-Net, which concatenates multilevel feature maps to enhance accuracy by combining low-resolution contextual information with high-resolution spatial details. This architecture has become a benchmark in biomedical image segmentation. Building upon U-Net, Ref. 18 integrated residual connections into the encoder–decoder framework, replacing standard neural units with residual blocks to facilitate deeper network training. Further advancing this paradigm, Ref. 19 proposed DeepLabV3, which employs dilated convolutions with varying dilation rates to expand receptive fields and capture multiscale contextual features. Regarding automatic segmentation of IVOCT vessel walls, Yong et al.^20^ proposed a linear regression convolutional neural network for fully automated coronary lumen segmentation in intravascular optical coherence tomography. Balaji et al.^21^ developed a novel capsule-based deep learning approach characterized by low memory consumption, fast inference speed, and maintained segmentation quality. However, it should be noted that these models require substantial computational resources and have only demonstrated good performance on small datasets, struggling to achieve reliable segmentation under various imaging conditions.^22^ With the advancement of deep learning, recent attention mechanisms and feature fusion methods have significantly enhanced traditional networks. Huang et al.^23^ proposed a multiscale feature fusion deep segmentation network with attention mechanisms for IVOCT lumen contour detection. By incorporating residual networks and attention mechanisms, their approach improved global feature extraction capabilities while achieving better robustness and accuracy. Wu et al^24^ developed a new deep-learning-based segmentation method based on a co-training architecture with an integrated structural attention mechanism. By utilizing features from three consecutive slices and incorporating spatial-channel attention modules, their method enhanced segmentation accuracy. Nevertheless, the performance of deep learning segmentation models fundamentally depends on IVOCT image quality. When dealing with poor-quality images or those with significant blood artifacts, the automatic segmentation accuracy inevitably decreases substantially due to missing image information, making reliable segmentation challenging. Therefore, handling interference factors such as blood artifacts remains an open challenge.

All aforementioned segmentation models rely solely on spatial features from individual images, neglecting the temporal characteristics inherent in IVOCT scanning sequences. For temporal feature extraction, Shi et al^25^ proposed the convolutional LSTM network (ConvLSTM) for precipitation forecasting. By extending fully connected LSTM (FC-LSTM) with convolutional structures in both input-to-state and state-to-state transitions, ConvLSTM can better capture spatiotemporal correlations in image sequences for short-term forecasting. In recent years, ConvLSTM has been increasingly applied to medical image analysis. Wong et al^26^ implemented brain image segmentation using bi-directional convolutional LSTM combined with U-Net, whereas Almiahi et al.^27^ proposed a novel ConvLSTM-based U-net architecture for improved brain tumor segmentation, both achieving superior results. However, no temporal feature extraction network has been specifically optimized for IVOCT imaging. To bridge this gap, we introduce ConvLSTM into IVOCT lumen segmentation, leveraging its capability to extract inter-frame spatiotemporal features from pullback sequences. These temporal features are then fused with spatial features extracted by advanced encoders, enabling multimodal fusion that compensates for artifacts and improves segmentation accuracy.

In this paper, we propose a novel deep learning segmentation network temporal residual U-Net (TR-Unet) that incorporates spatiotemporal features from IVOCT image sequences, representing the first attempt to introduce temporal characteristics into IVOCT lumen segmentation. We employ a residual neural network (ResUNet)^18^ to accelerate model convergence and enhance global feature extraction capabilities, where direct shortcut connections between inputs and outputs alleviate gradient vanishing issues. Furthermore, we utilize ConvLSTM to extract spatiotemporal features from preceding images, operating in parallel with ResU-Net’s encoder layers. Through attention mechanisms that fuse temporal and spatial information, our model achieves reliable segmentation of regions affected by blood artifacts or imaging blurring, effectively mitigating segmentation errors caused by missing information. Comparative experiments were conducted between the proposed model and traditional UVCR segmentation methods as well as nontemporal mainstream neural networks with similar encoder–decoder architectures (ResUnet, DeepLabV3). In addition, segmentation performance under varying temporal sequence lengths was validated. By processing 2451 clinical images, the results demonstrate that our temporal-aware method achieves superior performance across multiple metrics, including Dice coefficient = 98.54%, Jaccard similarity (JS) = 97.17%, and recall = 98.26%. More importantly, we specifically tested the model’s robustness using 216 images with severe blood artifacts. Experimental results confirm that our approach maintains significant performance advantages under challenging interference conditions.

## 2 Methods

**Fig. 2:**
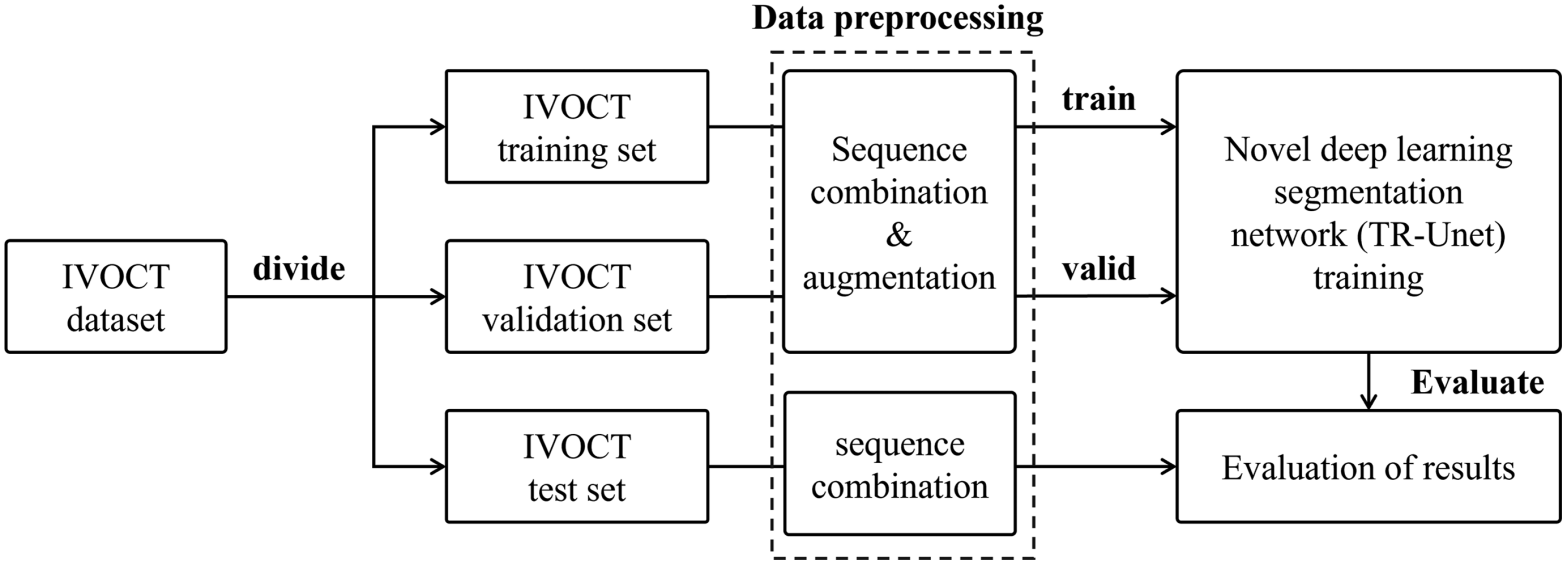
Data processing flowchart for the lumen segmentation method in IVOCT.

The workflow of the proposed lumen segmentation method in IVOCT is shown in Fig. 2. The dataset is partitioned into three subsets: a training set, a validation set, and a test set. The training set is used for model training, the validation set monitors training progress and guides hyperparameter selection, whereas the test set computes objective evaluation metrics to assess model performance. Subsequently, each batch of data undergoes preprocessing steps, including sequence grouping and image augmentation. The pre-processed image sequences are fed into the spatiotemporal feature network for training and validation, with final results analyzed through objective metric evaluation.

**Fig. 3:**
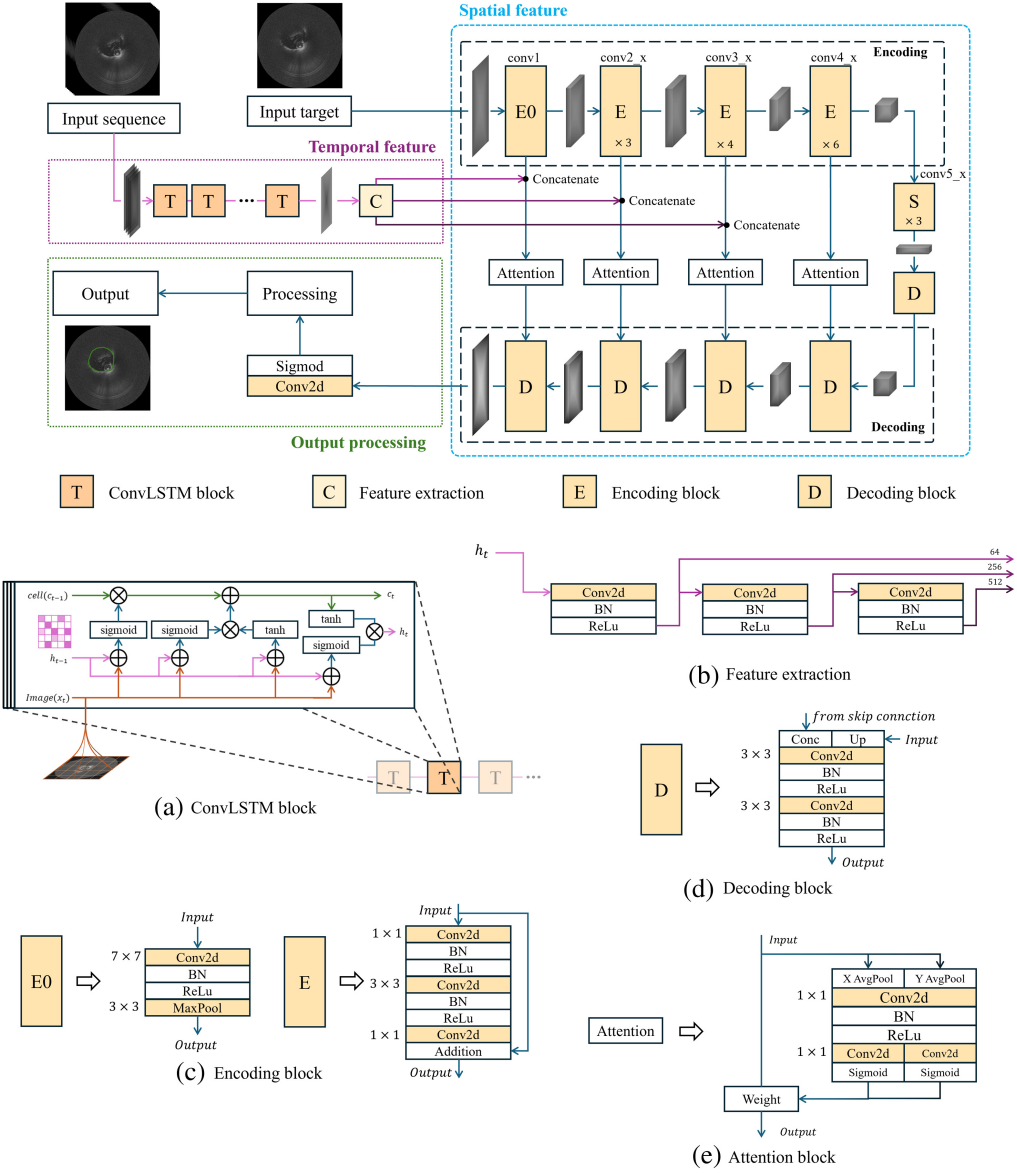
Overview of the proposed TR-Unet model for lumen segmentation. (a) ConvLSTM block. (b) Feature extraction. (c) Encoding block. (d) Decoding block. (e) Attention block.

Detailed architecture of the proposed TR-Unet model is shown in Fig. 3, which comprises three core components: temporal feature extraction, spatial feature extraction, and result processing. First, a temporally ordered image sequence is processed through temporal networks to capture spatiotemporal features, whereas encoder layers simultaneously extract spatial features from the target segmentation image. The extracted features from both branches are fused, and an attention mechanism prioritizes critical spatiotemporal information before transmitting it to the decoder via skip connections. The combined features are refined into an output weight map, from which the final segmentation result is derived through mathematical post-processing and optimization. Detailed implementations of each component will be analyzed in subsequent sections.

### 2.1 Data Preprocessing

The IVOCT system based on a catheter pullback imaging mechanism achieves continuous 3D vascular lumen imaging through constant-speed axial pullback of the probe during data acquisition. This imaging modality exhibits distinct spatiotemporal coupling characteristics:

• Temporal dimension: Due to physical constraints between the sampling rate and pullback speed, adjacent image frames maintain strict temporal sequential relationships.
• Spatial dimension: The spatial sampling interval along the vascular longitudinal axis is determined jointly by the pullback speed and frame rate, ensuring coherent anatomical structure representation.

Such spatiotemporal coupling results in locally significant spatial continuity of vascular wall morphological features within image sequences. Therefore, we group images adjacent to the segmentation target into sequential batches, forming 4D tensors (with added temporal dimensions) as fundamental data units.

**Fig. 4:**
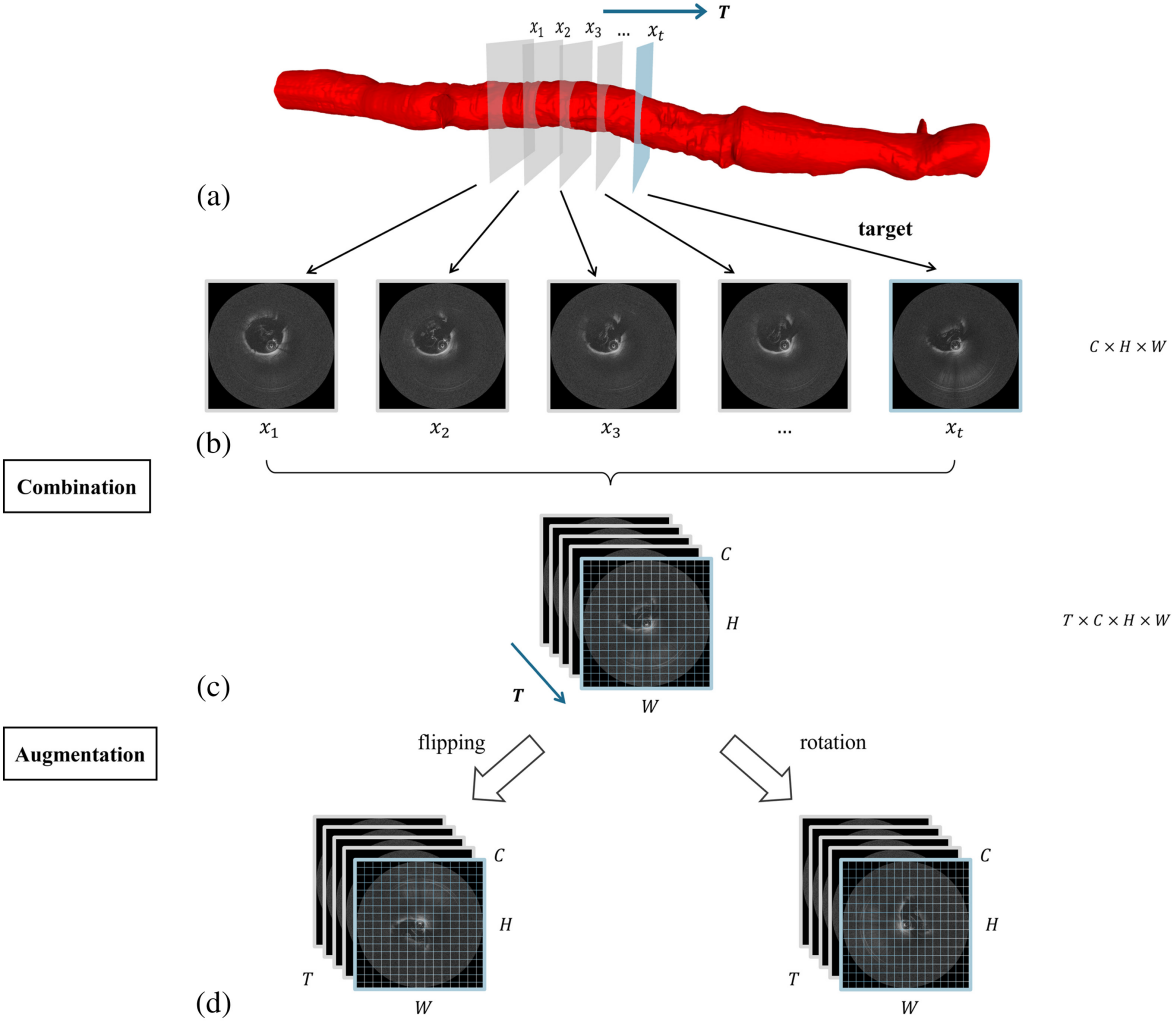
Data preprocessing pipeline for lumen segmentation. (a) Schematic of IVOCT catheter pullback imaging. Continuous image acquisition along the pullback direction (T-axis) establishes temporal relationships among sequential frames. (b) An IVOCT pullback sequence containing cross-sectional images at multiple longitudinal positions. $x_{t}$ denotes the target image for segmentation. (c) Temporal sequence composition. Input units for the temporal feature network are structured as 4D tensors with dimensions (C,H,W,T), where: C is the number of channels, H and W are the height and width of each individual frame, T is the temporal dimension (sequence length), stacked along the pullback direction. (d) Data augmentation strategies, including horizontal/vertical flipping and rotation. Identical augmentation operations were applied to entire data units to preserve spatiotemporal continuity.

Given the limited size of acquired IVOCT datasets, small training samples may lead to suboptimal model performance. To address this, we perform image augmentation during preprocessing, including horizontal/vertical flipping and rotation to diversify lumen orientations. Crucially, identical augmentation operations are applied to entire data units to preserve spatiotemporal continuity. The complete preprocessing pipeline is illustrated in Fig. 4.

### 2.2 Temporal Feature Extraction

The catheter pullback mechanism of IVOCT imaging creates a natural spatiotemporal coupling within the image sequence. Adjacent frames exhibit highly continuous anatomical structures due to the continuous physical acquisition process. The temporal features extracted by our model essentially leverage this continuity to learn the morphological evolution of the vascular wall along the longitudinal (pullback) direction. Specifically, the model learns not conventional “motion” but establishes spatial correlations across frames through sequential context. This enables the propagation of confident segmentation information from unambiguous frames to those corrupted by interference such as blood artifacts. This mechanism allows the model to use the vessel’s own structure as a guide to counteract noise and artifacts during imaging.

We employ ConvLSTM as the fundamental unit for spatiotemporal dependency feature extraction. Long short-term memory (LSTM), a specialized recurrent neural network (RNN) architecture, has demonstrated exceptional capabilities in modeling sequential dependencies across various domains.^28^^,^^29^ Compared with traditional RNNs, LSTM introduces three unique gating mechanisms—the input gate, forget gate, and output gate—alongside a cell state unit, enabling superior handling of long-term dependencies in sequences. However, conventional LSTM models cannot effectively process spatial information as they flatten inputs into one-dimensional vectors through fully connected (FC) operations, thereby discarding spatial encoding. To address this limitation, ConvLSTM was proposed as a spatially aware sequential model,^25^ replacing FC layers with convolutional operations to jointly capture spatiotemporal patterns. This advancement has led to its widespread adoption in meteorology, engineering, and medical imaging.^30^^–^^32^ We specifically chose ConvLSTM over alternative architectures for its superior suitability to our task. Compared with 3D CNNs,^33^ which require a new set of parameters for each additional temporal step, ConvLSTM’s recurrent nature provides a parameter-efficient way to model variable-length sequences. Furthermore, unlike transformers^34^ that demand massive datasets and excel at long-range dependencies, ConvLSTM is data-efficient and is ideally suited for capturing the strong short-term temporal continuity inherent in IVOCT pullbacks, which is precisely in lumen segmentation.

The ConvLSTM architecture retains the core structure of standard LSTM but replaces fully connected layers with convolutional operations. Its operational mechanism can be decomposed into spatiotemporally coupled gating processes as follows:

#### 2.2.1 Forget gate

Responsible for modeling historical memory attenuation, mathematically can be expressed as

$$f_{t}=\sigma (W_{xf}*x_{t}+W_{hf}*h_{t-1}+W_{cf}⊙c_{t-1}+b_{f}),$$ (1)

where * denotes the convolution operator, ⊙ represents the Hadamard product, $f_{t}$ is the forget gate, W denotes convolutional kernels, and x, h, c, and b correspond to input, hidden state, cell state, and bias, respectively. Spatially sensitive gating weights generated via convolutional operations are quantized by the sigmoid activation σ, adaptively eliminating morphological noise induced by catheter motion while preserving anatomically critical regions in $c_{t-1}$.

#### 2.2.2 Input gate

Implements feature updates through dual-path convolutional operations:

$$i_{t}=\sigma (W_{xi}*x_{t}+W_{hi}*h_{t-1}+W_{ci}⊙c_{t-1}+b_{i}),$$ (2)

$$g_{t}=\tanh(W_{xc}*x_{t}+W_{hc}*h_{t-1}+b_{c}).$$ (3)

Equation (2) regulates the fusion intensity of current frame information, whereas Eq. (3) captures local spatial patterns of vascular cross-sections via hyperbolic tangent activation. Their Hadamard product $i_{t}⊙g_{t}$ achieves incremental feature updates, ensuring spatiotemporal continuity in lumen boundary evolution.

#### 2.2.3 Cell state update

Cell state update follows the “selective forgetting-dynamic enhancement” principle:

$$c_{t}=f_{t}⊙c_{t-1}+i_{t}⊙g_{t}.$$ (4)

This dynamic equilibrium explicitly enforces topological constraints across vascular structures in sequential frames, effectively suppressing morphological abruptness caused by catheter pullback.

Output gate: generates hidden states through spatial modulation:

$$o_{t}=\sigma (W_{xo}*x_{t}+W_{ho}*h_{t-1}+W_{co}⊙c_{t}+b_{o}),$$ (5)

$$h_{t}=o_{t}⊙\tanh(c_{t}).$$ (6)

Equations (5) and (6) nonlinearly map the cell state into feature space, producing high-level semantic features fused with spatiotemporal context. These features provide robust state representations for downstream segmentation networks.

The temporal feature extraction layer employs cascaded ConvLSTM modules shown in Fig. 3(a), each containing multiple ConvLSTM cells. The output spatiotemporal features undergo resolution adjustment via a multi-scale convolutional encoder–decoder shown in Fig. 3(b) and are concatenated with spatial feature maps through skip connections, achieving synergistic optimization of spatiotemporal information for lumen segmentation. This architecture not only mitigates gradient anomalies in long-sequence training through convolutional gating mechanisms but, more importantly, enhances robustness to pullback motion and physiological motion artifacts, effectively preserving vascular morphological continuity, thereby enabling accurate segmentation of the lumen structure in complex scenarios.

### 2.3 Spatial Feature Extraction

Accurate lumen segmentation in IVOCT images depends not only on temporal information from preceding frames but also critically on the spatial features of the target segmentation image. In light of this, we design a spatial feature fusion network inspired by the residual UNet (ResUNet) architecture.^18^ ResUNet, a variant of the ResNet framework first proposed in 2015,^35^ leverages residual connections to facilitate network training. Building upon the U-Net foundation,^17^ ResUNet integrates residual connections within both encoder and decoder modules, incorporating skip connections across layers to mitigate vanishing gradient issues and preserve low-frequency features and semantic information.

As shown in Fig. 3, our vascular segmentation network adopts a modified ResUNet architecture. The encoder path follows the ResNet-50^35^ residual topology, utilizing bottleneck residual blocks as fundamental units. These blocks stack convolutional layers (Conv-BN-ReLU) with skip connections to construct a deep feature extractor. This design alleviates gradient vanishing while enhancing the network’s ability to discern subtle vascular wall textures. Traditional global pooling is replaced with stride 2 and size 3×3 convolutional layers for spatial downsampling. Unlike nontrainable pooling operations, convolutional downsampling adaptively retains critical spatial information of vascular anatomy through parameter optimization. The decoder employs cascaded transposed convolutions and feature fusion modules, progressively upsampling to reconstruct high-resolution lumen segmentation masks. Specifically, the decoder path consists of four symmetric upsampling modules. Each module first performs a strided transposed convolution (kernel_size = 2, stride = 2) to upsample the feature maps. This operation is then combined with skip connections, followed by a 3×3 convolution and ReLU nonlinear activation. Through this progressive process, the spatial resolution of the feature maps is incrementally restored to match the original input dimensions. Detailed encoder and decoder modules are depicted in Figs. 3(c) and 3(d). Notably, the E0 module consists solely of a 7×7 convolutional layer and a 3×3 max-pooling layer. Post-convolution operations include batch normalization and ReLU activation to extract low-level features and enhance nonlinear representation, consistent with ResNet-50. Similarly, convolutional layers in D0 are adjusted to ensure dimensional compatibility. For a generic encoder, the residual connection can be formulated as

y=F(x)+x, (7)

where x   is the input and F(x) denotes the transformed output through the layer.

To comprehensively exploit both temporal and spatial information during decoding, temporal features are concatenated with skip-connected spatial features, and the coordinate attention (CA) mechanism^36^ is introduced to enhance the model’s perception of heterogeneous feature representations. As illustrated in Fig. 3(e), the CA module enhances features through coordinate information embedding and attention map generation. Global average pooling is independently applied along the height (H) and width (W) axes to generate orientation-sensitive descriptors

$$\left\{ \begin{array}{c} z_{c}^{h}(i)=\frac{1}{W}\sum_{j=1}^{W}X_{c}(i,j)\\ z_{c}^{w}(j)=\frac{1}{H}\sum_{i=1}^{H}X_{c}(i,j) \end{array},\right.$$ (8)

where $X_{c}$ denotes the input feature map at channel. The concatenated descriptors undergo a nonlinear transformation via convolutional layers and ReLU activation

$$f=\delta (F_{1}([z^{h},z^{w}])),$$ (9)

followed by spatial decomposition and sigmoid-activated attention weight generation

$$\left\{ \begin{array}{l} g^{h}=\sigma (F_{h}(f^{h}))\\ g^{w}=\sigma (F_{w}(f^{w})) \end{array}.\right.$$ (10)

The original features are adaptively recalibrated through element-wise multiplication

$$y_{c}(i,j)=x_{c}(i,j)\times g_{c}^{h}(i)\times g_{c}^{w}(j).$$ (11)

By decoupling attention, this mechanism captures long-range dependencies horizontally while preserving vertical positional awareness, synergistically enhancing feature representation for target structures through orientation-sensitive and position-aware attention maps.

## 3 Experiments and Results

### 3.1 Dataset

The dataset comprises 2451 IVOCT frames from diverse patients in the database of the Chinese PLA General Hospital (Beijing, China). These patients were selected from the IVOCT images database of Chinese PLA General Hospital (Beijing, China). The average age of the patients is 64 years, with a standard deviation of 8 years. This database was approved by the independent ethics committee of Chinese PLA General Hospital, Beijing, China (2017 ethical examine 035). These are divided into 1400 training samples, 550 validation samples, and the remainder for testing. Raw data were acquired using a commercial swept-source IVOCT system (F-1, Nanjing Forssmann Medical Technology Co., China). The IVOCT system is based on swept-source OCT, which operates at a central wavelength of 1310 nm with a spectral bandwidth of >80  nm, an axial resolution of <20  μm, and a lateral resolution of <100  μm. The A-scan rate is 100 kHz. The frame rate is ∼100  fps. The imaging field of view (FOV) has a scan diameter of <7  mm, and pullbacks were performed at a standardized speed of 18 mm/s over a length of 54 mm. Original images (500×1024  pixels) underwent polar coordinate transformation and resizing to 512×512 resolution. All manual lumen segmentation tasks are performed by doctors with extensive expertise in vascular segmentation.

### 3.2 Training Implementation

All experiments are conducted on a Windows 11 (64-bit) system with an NVIDIA RTX4060 GPU. Models are built using the PyTorch framework. For temporal sequence models, memory units are initialized with zero values and progressively trained. For residual networks, PyTorch’s official pre-trained weights are adopted, with encoder parameters fine-tuned during training. Model parameters are updated iteratively using training data, whereas validation sets guide hyperparameter selection. Each training batch undergo data augmentation, as described in Sec. 2.1.

The loss function employed is Dice loss, which quantifies similarity between samples:

$$L_{\text{dice}}=1-\frac{2\times |X\cap Y|}{\left|X|+|Y\right|},$$ (12)

where |X∩Y| denotes the intersection, |X| and |Y| represent element counts, and the doubled numerator ensures a [0,1] range. Dice loss demonstrates superior performance in addressing class imbalance issues, effectively alleviating the adverse effects caused by foreground-background (pixel area) imbalance in samples. This enhances the model’s sensitivity to underrepresented regions, particularly in scenarios with sparse target annotations.

Stochastic gradient descent (SGD) serves as the optimizer, leveraging mini-batches to reduce computational overhead. The update rule is

$$\theta_{t+1}=\theta_{t}-\eta \nabla_{\theta }L(\theta ),$$ (13)

where $\theta_{t}$ denotes parameters at iteration t, η is the learning rate, and $\nabla_{\theta }L(\theta )$ is the loss gradient. SGD’s inherent randomness helps escape local minima, whereas momentum and learning rate adjustments ensure efficient convergence.^37^ Table 1 summarizes the training configurations.

**Table 1:** Training hyper-parameters.

Parameter	Value
Epoch	100
Batch size	8
Learning rate	0.1
Loss function	Dice loss
Optimizer	SGD

### 3.3 Evaluation Metrics

Five metrics—Dice coefficient (Dice), accuracy (ACC), Jaccard similarity (JS), recall (RECALL), and precision (PRE)—are employed to evaluate segmentation performance. Their definitions are as follows:

I. Dice coefficient:
  $$\text{Dice}=\frac{2\times |A\cap B|}{\left|A|+|B\right|},$$ (14)
  where A and B represent the predicted segmentation and ground truth mask, respectively. |A∩B| denotes their overlapping area, whereas |A| and |B| are their total areas. This metric quantifies the similarity between predictions and labels.
II. Accuracy
  $$\mathrm{ACC}=\frac{\mathrm{TP}+\mathrm{TN}}{\mathrm{TP}+\mathrm{TN}+\mathrm{FP}+\mathrm{FN}},$$ (15)
  where TP is true positives. TN is true negatives, FP is false positives, and FN is false negatives, defined at the pixel level. Accuracy measures the overall correctness of predictions across all samples.
III. Jaccard similarity (JS)
  $$\mathrm{JS}=\frac{\left|A\cap B\right|}{\left|A\cup B\right|}.$$ (16)
  This metric evaluates the overlap ratio between predictions (A) and labels (B), where |A∪B| represents their union.
IV. Recall
  $$\text{Recall}=\frac{\mathrm{TP}}{\mathrm{TP}+\mathrm{FN}}.$$ (17)
  Recall assesses the proportion of actual positive pixels correctly identified, reflecting the model’s coverage of all target regions.
V. Precision (PRE)
  $$\text{Precision}=\frac{\mathrm{TP}}{\mathrm{TP}+\mathrm{FP}}.$$ (18)
  Precision measures the fraction of correctly predicted positive pixels among all predicted positives, indicating detection reliability.

### 3.4 Results

The proposed TR-Unet model is applied to lumen segmentation and compared against nontemporal mainstream medical segmentation models with similar encoder–decoder architectures—DeepLabV3^19^ and ResUnet^18^—as well as the traditional UVCR method.^16^ All baseline models adopt encoder–decoder frameworks utilizing ResNet-based encoders for hierarchical feature extraction but differ in feature sampling strategies. For instance, DeepLabV3 integrates dilated convolutions within its encoder–decoder structure to balance precision and computational efficiency by flexibly controlling feature resolution, whereas ResUnet incorporates residual connections into the U-Net backbone to preserve identity mapping capabilities, thereby preventing performance degradation with increasing network depth. Notably, these baseline models can be regarded as nontemporal variants of TR-Unet due to structural similarities. Besides, to investigate temporal sequence length impacts, experiments are conducted with sequence lengths of 2, 3, 5, and 10 frames for spatiotemporal feature extraction. In IVOCT pullback imaging, however, excessive sequence lengths (>5 frames) introduce noise from distant frames, where rapidly decaying spatiotemporal correlations disrupt gradient propagation and exacerbate overfitting, adversely affecting the segmentation of adjacent frames. Empirical results confirm optimal performance at a sequence length of three frames, and all reported data are obtained under this configuration.

**Fig. 5:**
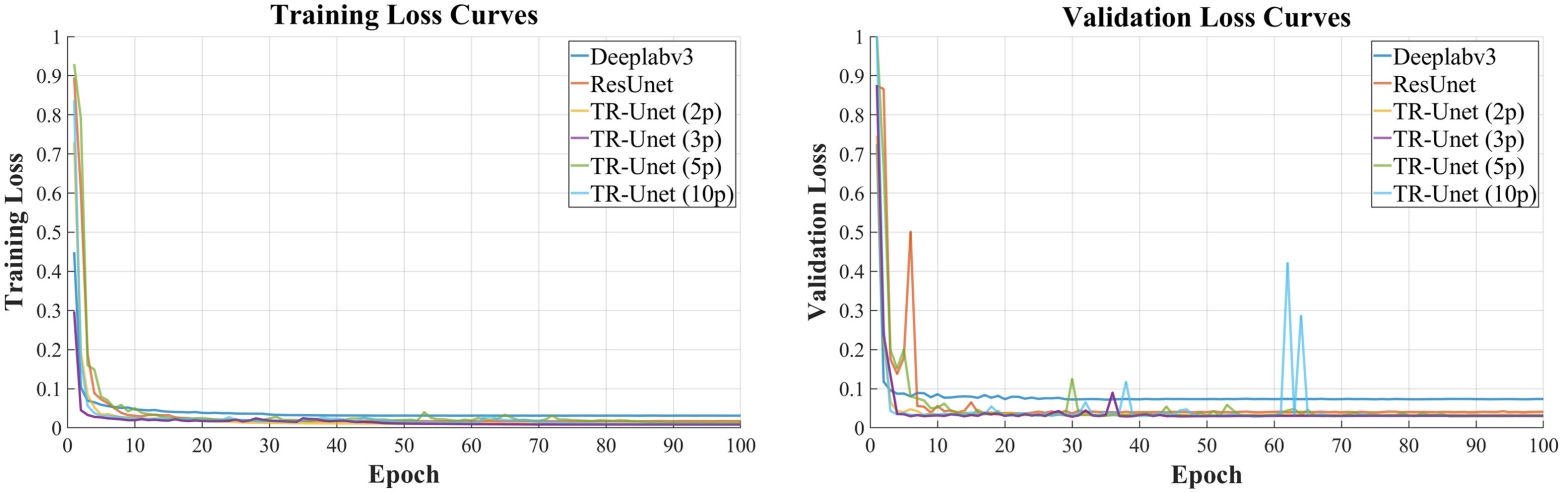
Training set and validation set loss versus epochs during the training process.

**Fig. 6:**
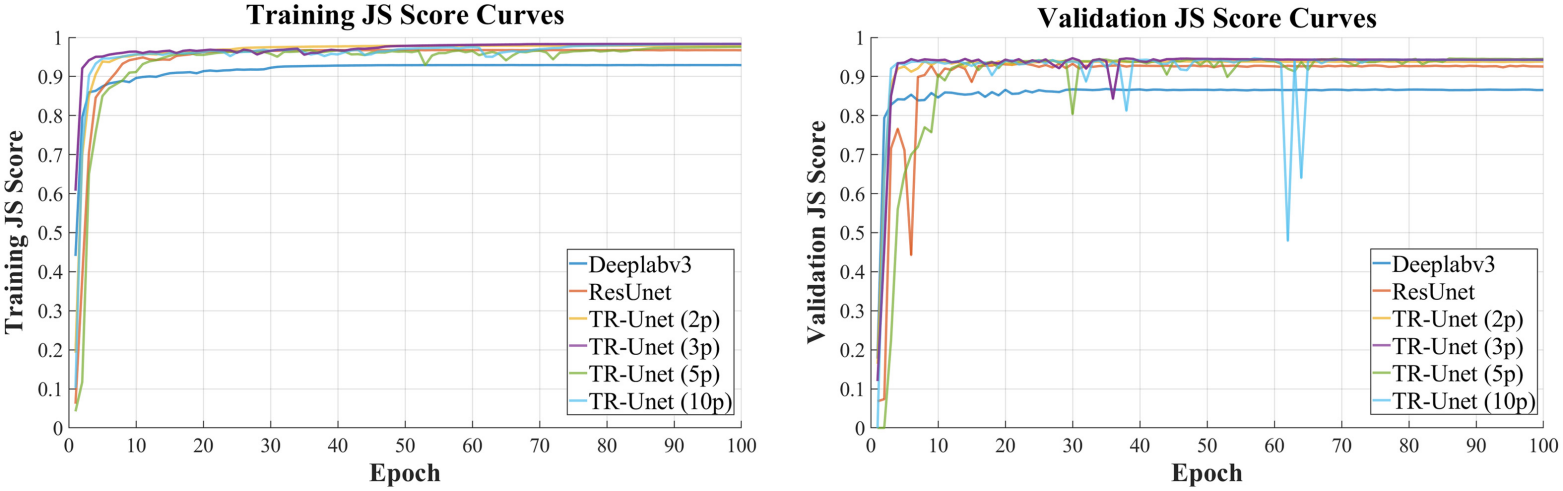
Training set and validation set Jaccard similarity (JS) versus epochs during the training process.

Figure 5 depicts the training loss curves of different networks. As the number of epochs increases, the loss gradually decreases, indicating that the models learn latent features from the training data and progressively optimize their parameters. The loss curves exhibit an initial sharp decline followed by a gradual plateau, suggesting convergence toward optimal solutions. The proposed spatiotemporal network TR-Unet demonstrates the fastest loss reduction and convergence, outperforming the two mainstream baseline models. Regarding temporal sequence length, with three-frame sequences, training stability is maintained, whereas sequences >5 frames exhibited overfitting. Figure 6 illustrates the training process using the Jaccard similarity (JS) coefficient, the most stringent evaluation metric. Although 3-frame and 10-frame sequences exhibit the fastest learning rates, longer sequences (>5 frames) show instability during training, further validating that excessive sequence lengths exacerbate overfitting. This finding aligns with the physical imaging process: although longer sequences provide more contextual information, they also span a longer vascular segment. Within this segment, physiological motions and catheter pullback inaccuracies can cause nonrigid deformation of the vessel between distant frames, introducing misalignment artifacts and motion artifacts. In addition, as training progresses, the model’s accuracy stabilizes, showing significant improvements over the mainstream segmentation models.

**Fig. 7:**
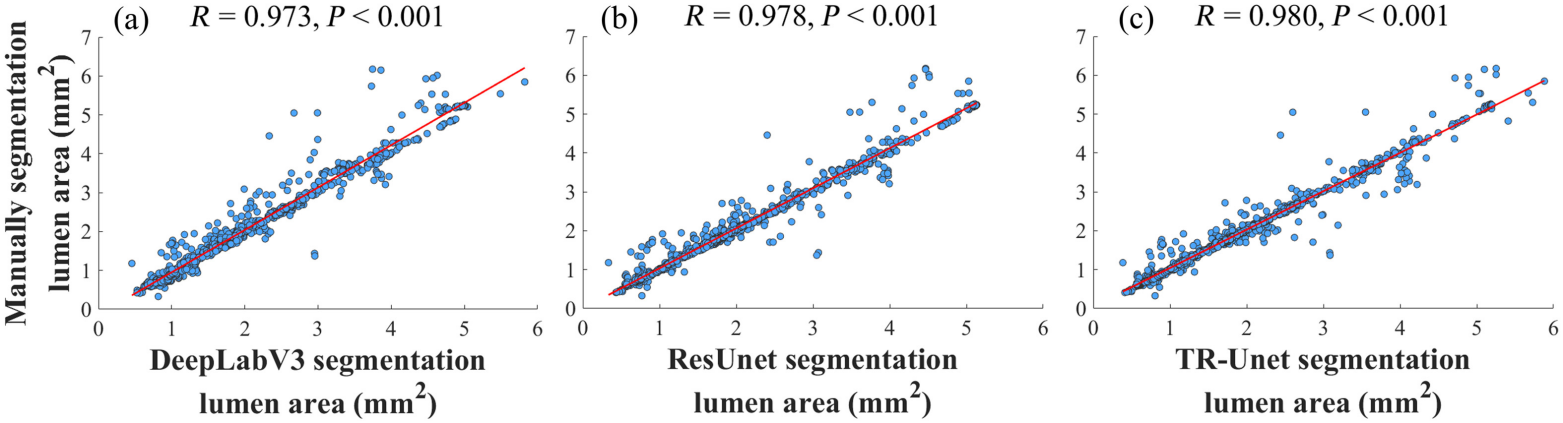
Correlation between automatically and manually segmented lumen areas: (a) DeepLabV3, (b) ResUnet, and (c) proposed method.

Table 2 summarizes segmentation results. Compared with the UVCR method, deep learning models significantly improve all metrics: Dice (+1.76%), ACC (+1%), JS (+3.41%), recall (+3.99%), and precision (+4.75%). Among deep models, TR-Unet achieves state-of-the-art performance with Dice = 98.54%, ACC = 99.64%, JS = 97.17%, and recall = 98.26%. To quantitatively evaluate segmentation accuracy, the correlation between automatically segmented lumen areas and manual segmentation results is calculated, as shown in Fig. 7. The proposed TR-Unet model achieves the highest Pearson correlation coefficient (R=0.980, p<0.001), confirming strong agreement with manual segmentation.

**Table 2:** Comparison of deep learning methods and traditional nondeep learning method in lumen segmentation.

	DICE (%)	ACC (%)	JS (%)	RECALL (%)	PRE (%)
DeepLabV3	97.56	99.35	95.28	97.31	97.62
ResUnet	98.46	99.61	97.02	97.97	**98.96**
UVCR method	96.78	98.64	93.76	94.27	94.21
Our method (TR-Unet)	**98.54**	**99.64**	**97.17**	**98.26**	98.82

Note: bold values represent the best performance for each metric.

**Fig. 8:**
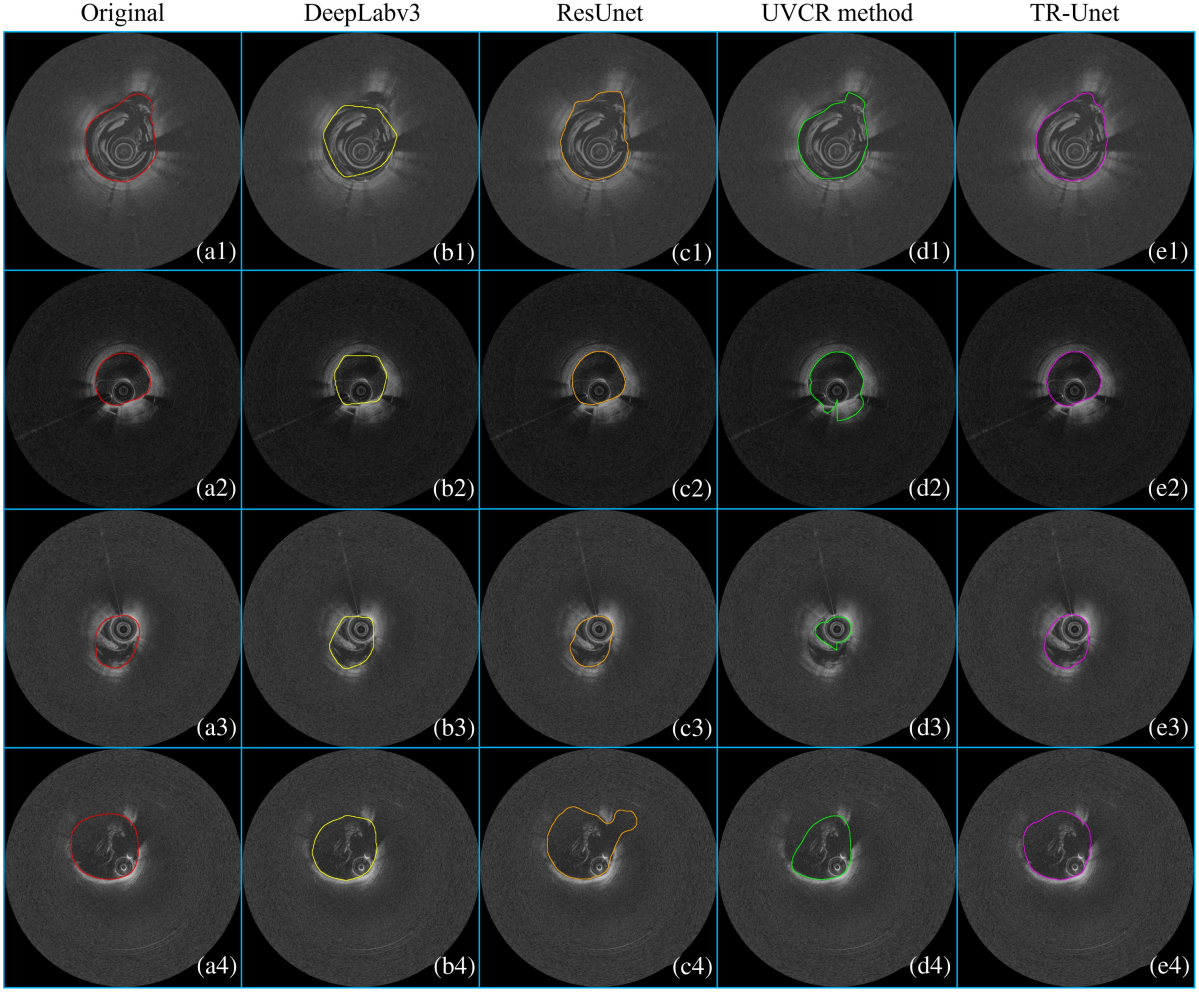
Lumen segmentation results of IVOCT images with severe blood artifacts. (a1)–(a4) Manual segmentation, (b1)–(b4) DeepLabV3 results, (c1)–(c4) ResUnet results, (d1)–(d4) UVCR method results, and (e1)–(e4) proposed method’s results.

More importantly, Table 3 evaluates robustness on 216 challenging images with severe blood artifacts. The proposed TR-Unet model surpasses other methods by leveraging temporal context to compensate for missing information, achieving improvements of 3.01% (Dice), 1.3% (ACC), 5.24% (JS), 2.15% (recall), and 2.06% (precision). Figure 8 demonstrates the lumen contour segmentation results of different models under severe blood artifacts. Panel (a) represents manual segmentation results, whereas panel (e) corresponds to the proposed TR-Unet’s outputs. The proposed TR-Unet model maintains robust segmentation performance despite blood artifacts, exhibiting high consistency with manual annotations. By contrast, other models [panels (b)–(d)] show significant deviations in specific cases, such as panels (b1)–(b3), (c1), (c4), and (d2)–(d4), indicating limited generalizability across diverse scenarios. These results confirm the superiority of our approach, particularly in severe blood artifacts.

**Table 3:** Comparison of deep learning methods and traditional nondeep method in blood artifact-affected lumen segmentation.

	DICE (%)	ACC (%)	JS (%)	RECALL (%)	PRE (%)
DeepLabV3	88.88	95.91	81.01	95.11	85.32
ResUnet	94.57	98.02	90.12	95.01	96.06
UVCR method	93.46	97.94	88.10	92.37	95.36
Our method (TR-Unet)	**97.58**	**99.32**	**95.36**	**97.16**	**98.12**

Note: bold values represent the best performance for each metric.

The computational efficiency and model complexity of all methods were evaluated on an NVIDIA RTX 4060 GPU to assess their practicality for clinical deployment. As summarized in Table 4, there exists a clear trade-off between segmentation accuracy and computational cost. As expected, the proposed model, with its additional temporal processing and attention mechanisms, has the highest computational complexity and the lowest inference speed among the compared models. This represents an ∼35% reduction in frame rate compared with the ResUNet backbone. However, for clinical relevance, the absolute inference speed is more critical than the relative reduction. Our model’s processing speed of 11.1 FPS translates to a capability of analyzing over 660 frames per minute, which is orders of magnitude faster than manual analysis. It has largely achieved the requirement for real-time analysis without the need for prior manual annotation by specialist clinicians. Therefore, the modest sacrifice in processing speed is strongly justified by the substantial gains in segmentation accuracy and robustness, particularly in challenging cases with artifacts, making the TR-Unet framework both effective and practicable for clinical use.

**Table 4:** Evaluation of computational efficiency and complexity under different models.

	Params (M)	MACs (G)	FPS	JS (%)
DeepLabV3	10.09	15.1	23.8	81.01
ResUnet	59.44	123.59	17.0	90.12
Our method (TR-Unet)	67.19	249.89	11.1	**95.36**

Note: bold values represent the best performance for each metric.

We also conducted a comprehensive ablation study to evaluate the contribution of each key component in the proposed architecture. The results are summarized in Table 5. The results clearly demonstrate that the CA module effectively refines the spatial features extracted by the ResUNet backbone, whereas the ConvLSTM module leverages temporal continuity. Their synergistic combination achieves the highest evaluation scores, confirming that each component plays a unique and complementary role in the success of the final model.

**Table 5:** Ablation study on the effectiveness of modules in blood artifact-affected IVOCT images.

	Params (M)	MACs (G)	DICE (%)	JS (%)
-ConvLSTM -CA (ResUnet)	59.44	123.59	94.57	90.12
-CA	59.70	123.63	95.28	91.09
-ConvLSTM	66.93	249.86	97.23	94.71
Our method (TR-Unet)	67.19	249.89	**97.58**	**95.36**

Note: bold values represent the best performance for each metric.

## 4 Discussion

Both traditional lumen segmentation methods and existing deep learning approaches rely solely on spatial information from the current image, making segmentation results inevitably susceptible to artifacts such as blood interference. This study pioneers the integration of temporal dimensions into IVOCT lumen segmentation, proposing a deep learning framework that leverages dual spatiotemporal information.

Traditional methods such as the UVCR method partially mitigate blood artifacts through conditional judgments and prior knowledge yet lack generalizability. Adjusting parameters for different patients or imaging devices limits their practicality. Existing deep learning models, constrained by spatial features alone, effectively exclude blood artifacts when vascular structures remain intact. However, due to IVOCT’s limited penetration depth, significant errors arise when complex artifacts cause partial lumen contour loss.

In evaluations, the proposed TR-Unet exhibited slight instability in normal image segmentation, manifesting as lower precision. This may stem from residual temporal noise in fused spatiotemporal features, where erroneous characteristics from preceding frames interfere with current predictions. Nevertheless, compared with the improvements in Dice coefficient, Jaccard similarity, and recall rate, this error can be considered negligible. Moreover, we are more concerned with the model’s adaptability to complex disturbances. In the segmentation of severely blood artifact-affected images, TR-Unet significantly outperforms other deep learning models because it leverages features from preceding images to compensate for the information loss caused by artifacts. This highlights its potential advantage in handling complex interference, particularly when sequential frames exhibit strong anatomical continuity. In summary, the proposed model demonstrates enhanced capability in learning detailed vascular contour features, contributing to smoother and more accurate lumen boundary delineation.

Regarding the general applicability of the proposed TR-Unet model, its core architecture is fundamentally designed to learn generalizable spatiotemporal features from IVOCT pullback sequences, rather than overfitting to specific hardware signatures. Therefore, the model holds significant potential for application to images acquired by other IVOCT instruments. The key prerequisite for such translation is the consistency of the basic imaging principles (i.e., catheter-based pullback acquisition) and the availability of corresponding annotated data for potential fine-tuning.

The proposed TR-Unet is a fully supervised framework that requires thousands of meticulously annotated images—a process that is both time-consuming and demands rare clinical expertise, posing a significant bottleneck for widespread clinical adoption. Future work will therefore focus on strategies to drastically reduce this annotation burden. The inherent temporal continuity of IVOCT pullback sequences provides an ideal foundation for this. Specifically, we plan to explore self-supervised pre-training by leveraging abundant unlabeled pullback data through pretext tasks, such as predicting the correct order of shuffled frames or reconstructing masked regions using contextual information from adjacent frames, or even enabling the model to learn to identify artifact locations itself, thereby adaptively incorporating temporal feature results to avoid noise introduction. This approach would allow the model to learn fundamental representations of vascular structure and temporal dynamics without manual annotation. Concurrently, we will investigate weakly-supervised learning paradigms that utilize simpler, less precise annotations to guide the segmentation of entire sequences, thereby optimizing the use of expert time. Through these directions, we aim to develop next-generation segmentation tools that are not only accurate but also practical and scalable for real-world clinical deployment.

## 5 Conclusion

This paper proposes TR-Unet, a deep learning model that integrates temporal sequence information of catheter pullback images with spatial features for IVOCT lumen segmentation—marking the first introduction of temporal characteristics into this task. By combining ConvLSTM’s temporal modeling capability, ResUnet’s spatial feature extraction, and Coordinate Attention’s cross-dimensional feature enhancement, TR-Unet achieves automated lumen segmentation with superior performance. Comparative experiments demonstrate its robustness against severe blood artifacts and potential advantages in complex scenarios. Future efforts will refine temporal architectures to further improve stability and generalizability.

## Data Availability

Data underlying the results presented in this paper are not publicly available at this time but may be obtained from the authors upon reasonable request.
